# The early impact of the COVID-19 pandemic on access to colorectal cancer treatment in Brazil: a spatial and interrupted time-series analysis

**DOI:** 10.1016/j.lana.2026.101406

**Published:** 2026-02-17

**Authors:** Luís Ricardo Santos de Melo, Júlio dos Santos Pereira, Matheus Santos Melo, Carlos Dornels Freire de Souza, Caíque Jordan Nunes Ribeiro, Carlos Anselmo Lima, Allan Dantas dos Santos

**Affiliations:** a Nursing Graduate Program, Federal University of Sergipe (UFS), São Cristóvão, Brazil; b Center for Research in Public Health, Federal University of Sergipe (UFS), São Cristóvão, Brazil; c Department of Pharmacy, Federal University of Sergipe (UFS), Lagarto, Brazil; d Department of Communicable Diseases, Ministry of Health (MS), Brazil; e Graduate Program in Tropical Medicine, University of Brasília (UNB), Brazil; f Faculty of Medicine, Federal University of Vale do São Francisco (UNIVASF), Brazil; g Health Sciences Graduate Program, Federal University of Sergipe (UFS), Aracaju, Brazil; h University Hospital, Federal University of Sergipe (UFS), Aracaju, Brazil; i Aracaju Cancer Registry, Aracaju, Brazil; j Department of Nursing, Federal University of Sergipe (UFS), Lagarto, Brazil

**Keywords:** Colorectal cancer, COVID-19, Treatment access, Health disparities, Interrupted time series

## Abstract

**Background:**

The coronavirus disease 2019 (COVID-19) pandemic has caused unprecedented disruptions in cancer care globally, disproportionately affecting access in low- and middle-income countries. In Brazil, data on how specific treatment modalities and delays have evolved during the pandemic remain limited. This study aims to evaluate the impact of the COVID-19 pandemic on access to colorectal cancer (CRC) treatment in Brazil, focusing on regional disparities in the time from diagnosis to treatment initiation across therapeutic modalities.

**Methods:**

We conducted a nationwide, population-based study using data from the Oncology Panel of the Brazilian Unified Health System (SUS). Therapeutic modalities included surgery, chemotherapy, radiotherapy, and combined chemoradiotherapy, categorized by treatment initiation intervals: within 30 days, 31–60 days, and over 60 days. The age-standardized treatment access rate (ASTAR) were defined as the rate of patients initiating treatment within each interval. Spatial analyses were performed to examine regional disparities across Health Regions using ASTAR percentage change. For temporal evaluation, we applied an interrupted time-series (ITS) analysis using monthly ASTAR and the impact of the COVID-19 pandemic on the treatment access rate (ITAR), defining the pre-pandemic period as January 2018–March 2020 and the post-pandemic period as beginning in April 2020. ITAR was estimated by comparing observed and expected delay rates in 2020.

**Findings:**

A total of 118,720 CRC treatment records were analyzed, of which 50.44% were male and 49.56% female. The most pronounced disruptions occurred in the Health Regions in the North, Northeast, and Midwest. ITS analysis revealed a significant level decrease by April 2020 for early access to all the treatments combined (−0.035; 95% CI: −0.091 to −0.022), chemotherapy (−0.040; 95% CI: (−0.096 to 0.018) and surgery (−0.040; 95% CI: (−0.096 to 0.018), while radiotherapy (0.002; 95% CI: 0.000–0.004) showed no meaningful level change. In 2020, a marked decline in access to early treatment was observed: surgery, chemotherapy and chemoradiotherapy initiated within 30 days decreased by 18% compared with the expected levels (0.82; 95% CI: 0.68–0.96). Conversely, the rate of radiotherapy within 30 days increased by 43% (ITAR = 1.43; 95% CI: 1.27–1.58). Overall, access rates remained below pre-pandemic trends until 2022.

**Interpretation:**

The COVID-19 pandemic substantially disrupted access to colorectal cancer treatment in Brazil, particularly for timely surgical and chemotherapeutic interventions. Regional inequalities were exacerbated, disproportionately affecting underserved areas with historical structural limitations. Although partial recovery occurred with some modalities, treatment rates remained below expected levels through 2022. These findings highlight the vulnerability of oncology care pathways during public health crises and underscore the need for resilient, regionally coordinated strategies to protect timely cancer treatment in future emergencies.

**Funding:**

None.

**Research in context**

**Evidence before this study**

Prior to this study, most of the available evidence on the impact of the COVID-19 pandemic on CRC treatment came from high-income countries and hospital-based cohorts. These studies largely focused on reductions in surgical procedures, screening and diagnosis, without evaluating the broader implications of health emergencies on access to treatment for public health systems. No previous population-based studies in Brazil or Latin America have comprehensively evaluated CRC treatment access across modalities via spatial analysis, interrupted time-series models, or delay-to-treatment metrics. To identify relevant literature, we searched PubMed and Scientific Electronic Library Online (SciELO) using the terms “colorectal cancer” AND “COVID-19” AND (“treatment” OR “access” OR “delay”), for articles published between 2020 and May 2025, in English, Portuguese, or Spanish. Studies were excluded if they addressed only screening or diagnosis without treatment outcomes, were single-center or small-cohort studies or did not report quantitative changes in treatment access or delays.

**Added value of this study**

Using national administrative data, our study evaluated age-standardized treatment access rates and delays across different therapeutic modalities from 2018 to 2022. By identifying the therapeutic modalities most affected and mapping regional disparities in access, our findings can inform public health policies aimed at strengthening the resilience and equity of oncology services. The observed delays and reductions in access underscore the need to expand early detection strategies, ensure continuity of care, and reinforce territorialized cancer networks, particularly in underserved areas. These insights are crucial for guiding recovery efforts and preparing health systems to safeguard timely cancer treatment in the face of future public health crises.

**Implications of all the available evidence**

This study reinforces the growing global evidence that the COVID-19 pandemic significantly disrupted cancer treatment pathways, particularly in middle-income countries such as Brazil, where structural inequities still exist. These findings emphasize the need for investment in robust, regionally coordinated cancer networks capable of withstanding systemic shocks. Strengthening early detection programs, improving data systems, and ensuring timely access to treatment should be central priorities. Furthermore, the methodology applied here can serve as a model for other countries seeking to monitor cancer care resilience and address geographic inequalities in access under both routine and emergency conditions.

## Introduction

Colorectal cancer (CRC) is the third most common cancer and the second leading cause of cancer-related mortality worldwide for both sexes, with an estimated 1.9 million new cases and 903,000 deaths in 2020.^1^ This burden has increased in low- and middle-income countries (LMICs), particularly in Latin America, where demographic aging, epidemiological transition, and persistent structural barriers to cancer care have contributed to rising incidence and mortality.^2^ In Brazil, national projections for 2023–2025 estimate an annual incidence of 21.1 new CRC cases per 100,000 inhabitants, with CRC-related mortality reaching 20,245 deaths annually.^3^ Despite the existence of a universal public health system, access to oncologic care shows substantial disparities across regions in the availability of treatment infrastructure and workforce capacity.^4^^,^^5^ These challenges are shared by several Latin American countries and are frequently reported in regional assessments of cancer care delivery, highlighting the vulnerability of CRC management in resource-constrained settings.^6^

The COVID-19 pandemic further exacerbated these pre-existing inequities. The reallocation of healthcare resources led to widespread delays in cancer care, including CRC treatment.^7^ While international oncology societies issued interim guidance to prioritize curative treatments and adapt therapeutic strategies during the pandemic,^8^^,^^9^ the implementation of such recommendations in South America was highly heterogeneous and often constrained by local health system capacity.^10^ In Brazil, the first COVID-19 case was reported in February 2020, followed by rapid escalation of infections and deaths. Public health responses were fragmented, resulting in sharp reductions in oncology procedures during the early pandemic waves, alongside declines in CRC screening and diagnoses.^11^, ^12^, ^13^

Timely access to cancer treatment is a critical determinant of outcomes, as delays in therapy initiation are associated with disease progression and reduced survival.^14^ In Brazil, Law No. 12.732/2012^15^ aims to address delays in treatment as it mandates that patients diagnosed with cancer must begin treatment within 60 days of receiving their diagnosis. By setting this deadline, the Brazilian government sought to improve access to timely care and reduce mortality rates associated with late treatment. For CRC, pandemic-related disruptions likely intensified these risks, amplifying existing system-level vulnerabilities and compromising patient-centered care.^16^ In this context, population-based surveillance and administrative data play a central role in monitoring treatment access and delays, offering the scale, continuity, and geographic coverage required to assess health system performance during large-scale public health crises.

This study aims to assess the impact of the COVID-19 pandemic on access to CRC treatment in Brazil using national administrative data. By integrating spatial and interrupted time-series analyses, we seek to identify regional disparities and modality-specific vulnerabilities related to time to treatment initiation, providing evidence to inform strategies for strengthening oncology care delivery.

## Methods

### Study design

We conducted a population-based study using spatial analysis and interrupted time-series (ITS) approaches. The analysis included aggregated data on CRC treatment in Brazil from 2018 to 2022, based on International Classification of Diseases (ICD-10) codes C18–C20.^17^ This period encompasses two pre-pandemic years and the subsequent pandemic years, allowing for the assessment of temporal changes associated with the COVID-19 health crisis. Ethics committee approval was not required, as the study used publicly available, aggregated data with no possibility of individual identification.

### Study setting

Brazil, the fifth largest and most populous country in the world, is administratively divided into 27 federative units and 5570 municipalities, grouped into five macroregions: North, Northeast, Southeast, South, and Midwest, each with distinct geographic and socioeconomic characteristics.^18^ For healthcare planning and analysis, municipalities are aggregated into 450 Health Regions, which constitute the unit of analysis in this study. These Health Regions are territorial entities designed to integrate health service delivery across socially and infrastructurally connected areas within the Unified Health System (SUS).^19^

### Data sources

Data on CRC treatment access were obtained from the Oncology Panel (*Painel Oncologia*), a national database maintained by the Department of Informatics of the Brazilian Unified Health System (DATASUS).^20^ This platform compiles information on the diagnosis and treatment of malignant neoplasms and is used to monitor compliance with Law No. 12.732/2012,^15^ which mandates the initiation of cancer treatment within 60 days of diagnosis. Data were retrieved for the therapeutic modalities of surgery, chemotherapy, radiotherapy and combined chemoradiotherapy, and were categorized by treatment initiation intervals: within 30 days, 31–60 days, and over 60 days. Annual and monthly population estimates, as well as shapefiles for Health Region boundaries, were obtained from the Brazilian Institute of Geography and Statistics (IBGE), using a geographic projection based on latitude and longitude.

### Statistical analysis

We first described the distribution of sociodemographic and clinical variables, including sex assigned at birth, age group, ICD-10 code (C18–C20), year of treatment, treatment modality, stage at diagnosis, and time from diagnosis to treatment initiation. The data were summarized in terms of absolute and relative frequencies for Brazil and its five macroregions.

The primary outcome of this study was the age-standardized treatment access rate (ASTAR) per 100,000 inhabitants. ASTARs were calculated for each Health Region using the direct standardization method based on the world standard population proposed by Segi–Doll.^21^^,^^22^ Age-specific rates were estimated in 5-year age groups (0–4, 5–9, …, 75–79, ≥80 years). The standardization allows robust temporal and spatial comparisons by accounting for differences in population age structures across regions and over time.

To evaluate the impact of the COVID-19 pandemic on access to CRC treatment in 2020, we calculated the percentage change (% change) in ASTAR by therapeutic modality and waiting time. The % change was calculated considering the relative difference between pre-pandemic average rate (2018–2019) and the post-pandemic average rate (2020). Positive values indicate an increase, and negative values a decrease in access relative to the baseline.

$$Percentage\;change\;\left(\%\right)=\left(\frac{Post-pandemic\;rate\;-\;Pre-pandemic\;rate}{Pre-pandemic\;rate}\right)X\;100$$

Analyses were stratified by Health Region. Results were visualized using choropleth maps divided into seven equal-interval categories based on % change values. Health Regions with missing treatment data for both pre- and post-pandemic periods were marked as 0%.

To determine whether changes in CRC treatment access following the onset of the COVID-19 pandemic deviated from pre-pandemic trends, we applied an ITS analysis. ITS analysis was conducted using two models: one using ordinary least squares (OLS) regression and the other using generalized least squares (GLS) regression. The GLS model was employed to adjust first-order autocorrelation and seasonality. The decision between the final OLS and GLS models was based on the degree of correction for autocorrelation and seasonality, measured by the formal Ljung–Box test and the ACF plot. Additionally, Akaike information criterion (AIC) was used to decide between the GLS and OLS models. When both models presented good parameters, the OLS model was chosen as the final model due to the greater simplicity of its parameters. The following regression model was used:

Yt=β0+β1∗T+β2∗Xlevel+β3∗Xtrend+harmonic(month;1;12)+εt

where *Yt* represents the monthly hand soap usage at time t; *β0* is the intercept; *β1* denotes the underlying pre-intervention temporal trend, with *T* indicating time in months; *β2* estimates the immediate change in level associated with the intervention, where *Xlevel* is a binary indicator coded 0 before and 1 after the intervention; *β3* captures the change in slope following the intervention, with *Xtrend* representing time elapsed since the intervention; *harmonic (month; 1; 12)* comprises harmonic terms included to account for annual seasonality; and *εt* denotes the random error term.

The outcome variable for ITS analysis was the monthly ASTAR. Monthly population estimates were generated through linear interpolation of annual data from 2018 to 2022 to ensure accurate rate calculations in the absence of official monthly estimates. The intervention point was defined as April 2020, considering that March was the month when the pandemic was established in Brazil. This timing reflects the period when disruptions to oncology services were expected to manifest in administrative treatment data. Statistical significance was set at p < 0.05.

We also calculated the impact of the COVID-19 pandemic on the treatment access rate (ITAR) as secondary outcome, defined as the ratio between the standardized rate observed in 2020 and the expected rate based on pre-pandemic trends. Confidence intervals (95% CIs) were estimated to assess statistical uncertainty, and the estimated model was created by a segmented regression model. ITAR lower than 1 means that a decrease in access to treatment occurred while greater than 1 indicates a rise, reflecting the pandemic's potential impact.

$$ITAR\;\left(\%\right)=\left(\frac{Observed\;age-adjusted\;access\;rate}{Estimated\;age-adjusted\;access\;rate}\right)X\;100$$

No missing data was identified in the variables used for the analyses. All treatment records extracted from the Oncology Panel of the Brazilian Unified Health System (SUS) contained complete information regarding treatment modality, time from diagnosis to treatment initiation. Therefore, no data imputation procedures were required, and all eligible records were included in the analyses.

Microsoft Excel^23^ was used for data tabulation, rate standardization, and calculation of percentage changes in treatment access. Spatial data processing and choropleth map generation were conducted in QGIS software (version 3.18.3).^24^ Interrupted time series analyses, including trend modeling and computation of the ITAR were performed in R software (version 4.2.2),^25^ which enabled advanced statistical modeling and temporal visualization.

### Role of the funding source

There was no funding source for this study.

## Results

### Sociodemographic and clinical characteristics

Between 2018 and 2022, a total of 118,720 patients with CRC received treatment in Brazil, with the majority concentrated in the Southeast (47.71%) and South (25.38%) regions. The sex distribution was slightly major in males (50.44%), although regional variations were observed. Most patients were aged 45–64 years (46.29%). Colon tumors (C18) predominated (62.12%), followed by the rectal tumors (C20, 33.49%). A progressive increase in treatment volume was noted over the study period, peaking in 2022 (22.10%). Chemotherapy was the most frequently administered modality (53.35%), followed by surgery (35.85%), with regional disparities in access. Advanced-stage diagnosis was common, with 24.57% of patients at stage III and 23.96% at stage IV. Regarding the timeliness of care, 43.27% of patients initiated treatment within 30 days of diagnosis, whereas 38.23% experienced delays exceeding 60 days; delayed initiation was particularly pronounced in North (48.41%) (Table 1).

**Table 1:** Sociodemographic and clinical characteristics of patients treated for CRC in Brazil, 2018–2022.

	North 3459 (2.92)	Northeast 19,668 (16.56)	Southeast 56,631 (47.71)	South 30,137 (25.38)	Central-West 8825 (7.43)	Brazil 118,720 (100.0)
**Sex**						
Male	1678 (48.50)	9373 (47.65)	28,556 (50.42)	15,805 (52.46)	4455 (50.47)	59,867 (50.44)
Female	1781 (51.50)	10,295 (52.35)	28,075 (49.58)	14,332 (47.54)	4370 (49.53)	58,853 (49.56)
**Age group**						
≤19	15 (0.43)	396 (2.01)	515 (0.91)	528 (1.75)	138 (1.56)	1592 (1.34)
20–44	601 (17.38)	3034 (15.42)	5452 (9.63)	3332 (11.06)	1246 (14.12)	13,665 (11.52)
45–64	1705 (49.29)	8872 (45.10)	26,633 (47.03)	13,403 (44.49)	4362 (49.43)	54,975 (46.29)
≥65	1138 (32.90)	7366 (37.47)	24,031 (42.43)	12,874 (42.70)	3079 (34.89)	48,488 (40.85)
**ICD-10**						
C18	1813 (52.41)	12,252 (62.30)	35,135 (62.06)	19,048 (63.22)	5522 (62.58)	73,770 (62.12)
C19	230 (6.65)	551 (2.80)	2568 (4.53)	1453 (4.81)	399 (4.52)	5201 (4.39)
C20	1416 (40.94)	6865 (34.90)	18,928 (33.41)	9636 (31.97)	2904 (32.90)	39,749 (33.49)
**Year of treatment**						
2018	566 (16.36)	3451 (17.54)	9967 (17.61)	5149 (17.09)	1424 (16.14)	20,557 (17.32)
2019	626 (18.10)	3830 (19.47)	11,330 (20.01)	5926 (19.67)	1685 (19.10)	23,397 (19.71)
2020	669 (19.35)	3900 (19.83)	11,234 (19.83)	6032 (20.01)	1757 (19.91)	23,592 (19.87)
2021	737 (21.30)	4231 (21.52)	11,947 (21.09)	6226 (20.66)	1794 (20.32)	24,935 (21.00)
2022	861 (24.89)	4256 (21.64)	12,153 (21.46)	6804 (22.57)	2165 (24.53)	26,239 (22.10)
**Treatment**						
Surgery	968 (27.98)	5786 (29.42)	21,261 (37.53)	11,342 (37.63)	3204 (36.31)	42,561 (35.85)
Chemotherapy	1975 (57.09)	11,158 (56.73)	29,013 (51.24)	16,206 (53.79)	4981 (56.44)	63,333 (53.35)
Radiotherapy	432 (12.49)	2239 (11.39)	5118 (9.04)	2185 (7.24)	476 (5.39)	10,450 (8.80)
Both (Chemo + Radiotherapy)	84 (2.44)	485 (2.46)	1239 (2.19)	404 (1.34)	164 (1.86)	2376 (2.00)
**Staging**						
0	29 (0.84)	261 (1.32)	1244 (2.20)	522 (1.73)	90 (1.02)	2146 (1.81)
1	63 (1.83)	284 (1.44)	960 (1.70)	986 (3.27)	104 (1.18)	2397 (2.02)
2	586 (16.94)	2852 (14.51)	6551 (11.57)	3125 (10.37)	874 (9.90)	13,988 (11.79)
3	974 (28.15)	5723 (29.09)	13,827 (24.42)	6641 (22.05)	2010 (22.78)	29,175 (24.57)
4	839 (24.26)	4762 (24.21)	12,788 (22.58)	7521 (24.95)	2543 (28.81)	28,453 (23.96)
Does not apply^a^	968 (27.98)	5786 (29.43)	21,261 (37.53)	11,342 (37.63)	3204 (36.31)	42,561 (35.85)
**Time until treatment**						
≤30 days	1162 (33.58)	7708 (39.21)	23,711 (41.88)	14,732 (48.89)	4068 (46.10)	51,381 (43.27)
31–60 days	623 (18.01)	3974 (20.21)	9611 (16.97)	6000 (19.91)	1748 (19.81)	21,956 (18.50)
>60 days	1674 (48.41)	7986 (40.58)	23,309 (41.13)	9405 (31.20)	3009 (34.09)	45,383 (38.23)

a Refers to patients treated with surgery. Surgery does not have staging information because this treatment is retrieved from the Hospital Information System (SIH), which does not contain this information.

### Spatial disparities

**Fig. 1:**
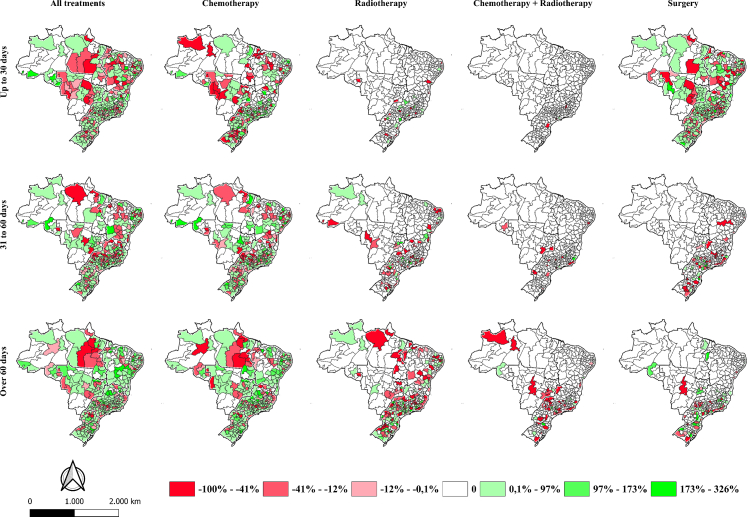
Spatial distribution of the percentage change for age-standardized treatment access rate for CRC across Brazil's Health Regions in 2020.

Regional disparities were observed in the percentage change of age-standardized treatment access rate for CRC across Brazil's Health Regions due to the COVID-19 health emergency, according to Fig. 1. For all the treatments combined, reductions exceeding 40% were concentrated in numerous regions of North and Midwest–most notably in central Pará and northern Mato Grosso, across all time intervals. In contrast, significant increases in access (up to 326%) were observed in parts of the South and Southeast, particularly for patients treated within 30 days. Chemotherapy was the most affected modality, with sharp declines (up to 100%) in the early treatment window (≤30 days) across several Health Regions in North, including western Amazonas. These reductions persisted at 31–60 day and >60-day intervals, although less intensely.

Radiotherapy access showed moderate changes overall, but notable declines were detected in selected Health Regions of the Southeast and South, particularly for treatments initiated within 60 days according to Fig. 1. Combined chemoradiotherapy access has experienced a decline especially in the Midwest, Southeast and South, with reductions exceeding 40% during the pandemic in patients initiating treatment after 60 days. Finally, surgical access presented a distinct pattern, with heterogeneous variation across the country. Several Health Regions in Northeast experienced considerable reductions, especially for early procedures (≤30 days). In contrast, substantial increases in timely surgical access were observed in South and Southeast states during the early treatment window (≤30 days).

### Interrupted time series

**Fig. 2:**
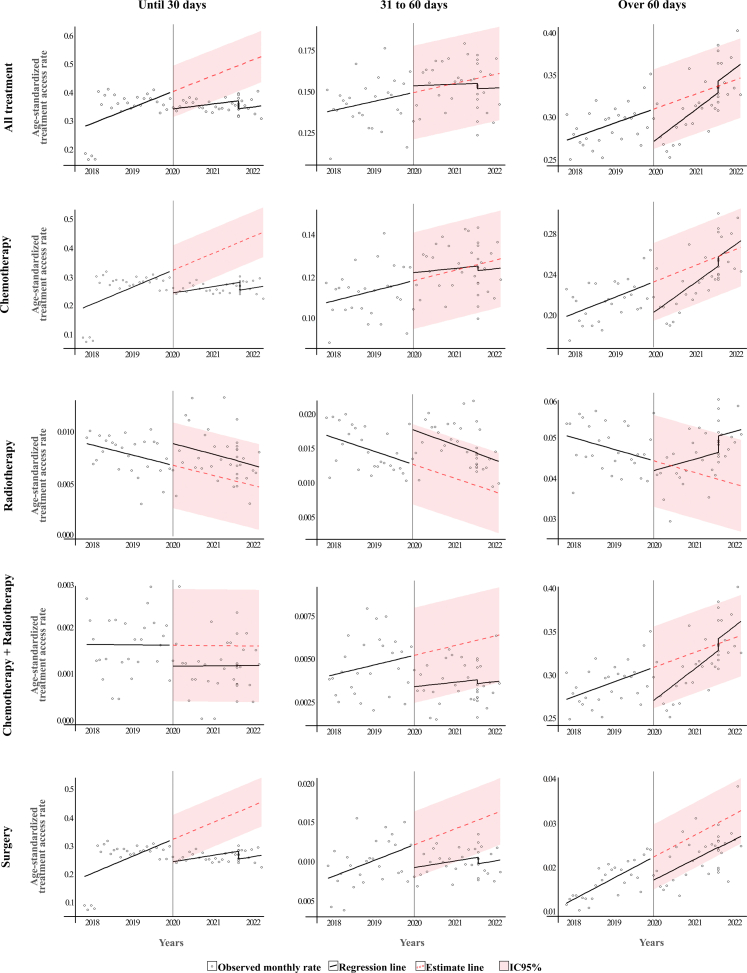
Interrupted time series analysis of the age-standardized treatment access rate for CRC in Brazil.

The interrupted time-series (ITS) analysis demonstrated that the COVID-19 pandemic produced its most pronounced effects on early treatment initiation (≤30 days) and on delayed treatment (>60 days), with heterogeneous patterns across treatment modalities. According to Table 2 and Fig. 2, for treatments initiated within 30 days, a significant immediate level decrease was observed in April 2020 for all treatments combined (estimate: −0.035; 95% CI: −0.091 to 0.022), corresponding to an absolute reduction of 3.5% in ASTAR. Similar declines were identified for chemotherapy and surgery (both −0.040; 95% CI: −0.096 to 0.018), indicating a 4.0% drop in early access for these modalities. In contrast, radiotherapy showed a small but significant positive level change (0.002; 95% CI: 0.000–0.004).

**Table 2:** Interrupted time series analysis of age-standardized treatment access rate for colorectal cancer in Brazil.

Time until treatment	Variables	All treatment		Chemotherapy		Radiotherapy		Chemotherapy + Radiotherapy		Surgery	
		Estimate	95% CI	Estimate	95% CI	Estimate	95% CI	Estimate	95% CI	Estimate	95% CI
≤30 days	Intercept	0.117∗∗∗	(0.075–0.159)	0.027	(−0.028 to 0.081)	0.013∗∗∗	(0.011–0.014)	0.002∗∗∗	(0.002–0.002)	0.0269	(−0.028 to 0.081)
	Trend before COVID-19	0.005∗∗∗	(0.003–0.005)	0.005∗	(0.003–0.006)	−0.000∗∗∗	(−0.000 to −0.000)	−0.000^a^	(−0.000 to 0.000)	0.005∗	(0.003–0.006)
	COVID-19	−0.035	(−0.091 to 0.022)	−0.040	(−0.096 to 0.018)	0.002∗	(0.000–0.004)	−0.000^a^	(−0.000 to 0.000)	−0.040	(−0.096 to 0.018)
	Post-pandemic start	−0.004	(−0.072 to 0.018)	−0.004	(−0.008 to 0.001)	0.000^a^	(−0.000 to 0.000)	0.000^a^	(−0.000 to 0.000)	−0.004	(−0.008 to 0.001)
31–60 days	Intercept	0.135	(0.131–0.139)	0.105	(0.101–0.108)	0.019∗∗∗	(0.017–0.020)	0.005∗∗∗	(0.004–0.006)	0.006	(0.005–0.006)
	Trend before COVID-19	0.002	(0.000–0.0003)	0.000	(0.000–0.003)	−0.000∗∗	(−0.001–0.000)	−0.000^a^	(−0.000 to 0.000)	0.000	(0.000–0.000)
	COVID-19	0.008	(0.001–0.014)	0.007	(0.002–0.013)	0.003∗	(0.000–0.006)	−0.000^a^	(−0.002 to 0.000)	−0.002	(−0.003 to 0.000)
	Post-pandemic start	−0.000	(−0.001 to 0.00)	−0.000	(−0.000 to 0.001)	−0.000	(−0.000 to 0.000)	0.000^a^	(−0.000 to 0.000)	−0.000	(−0.000 to 0.000)
>60 days	Intercept	0.195	(0.185–0.204)	0.136	(0.123–0.143)	0.040∗∗∗	(0.036–0.043)	0.194	(0.185–0.204)	0.009∗∗∗	(0.007–0.010)
	Trend before COVID-19	0.002	(0.002–0.002)	0.002	(0.002–0.002)	0.000∗∗∗	(0.000–0.000)	0.002	(0.002–0.002)	0.000∗∗∗	(0.000–0.000)
	COVID-19	−0.045	(−0.061 to 0.029)	−0.033	(−0.045 to 0.020)	−0.009∗∗∗	(−0.015 to 0.004)	−0.045	(0.061–0.029)	−0.002^a^	(−0.005 to 0.000)
	Post-pandemic start	0.00	(−0.000 to 0.001)	0.00	(−0.003 to 0.007)	0.000^a^	(−0.000 to 0.000)	0.000	(−0.000 to 0.001)	0.000∗	(0.000–0.000)

p-value: ∗<0.05; ∗∗<0.01; ∗∗∗<0.001.

a No significant (p > 0.05).

Among treatments initiated between 31 and 60 days, Table 2 and Fig. 2 shows no clinically meaningful level changes for most modalities. Pre-pandemic trends were largely stationary, and both level and slope changes after the intervention remained minimal. In other hand, for treatments initiated after 60 days, the pandemic was associated with significant negative level changes for all treatments combined (−0.045; 95% CI: −0.061 to −0.029), chemotherapy (−0.033; 95% CI: −0.045 to −0.020), and combined chemoradiotherapy (−0.045; 95% CI: −0.061 to −0.029), corresponding to absolute reductions of 3.3%–4.5% in delayed treatment access as seen in Table and Fig. 2.

### Impact of COVID-19 on treatment access rate (ITAR)

The ITAR analysis corroborated the ITS findings and allowed direct comparison between observed and expected access levels during 2020 as seen in Table 3. For treatments initiated within 30 days, ITARs were consistently below 1.0 for chemotherapy, surgery, and combined treatments (all ITAR = 0.82; 95% CI: 0.68–0.96), indicating a relative reduction of approximately 18% compared with expected levels. When considering all treatments combined, early access declined by 12%, reflecting clear underperformance of the system in delivering timely care during the first pandemic year. In contrast, radiotherapy exhibited an ITAR of 1.43 (95% CI: 1.27–1.58), corresponding to a 43% increase above expected levels, suggesting compensatory expansion.

**Table 3:** Impact of the COVID-19 pandemic on the treatment access rate (ITAR) across colorectal cancer treatment modalities.

Time until treatment									
Treatment modalities	Up to 30 days			31–60 days			>60 days		
	ITAR	95% CI	Relative change (%)	ITAR	95% CI	Relative change (%)	ITAR	95% CI	Relative change (%)
All treatment	0.88	(0.79–0.98)	12% (↓)	1.03	(0.99–1.06)	0.3% (⥈)	0.84	(0.82–0.87)	16% (↓)
Chemotherapy	0.82	(0.68–0.96)	18% (↓)	1.04	(1.01–1.08)	0.4% (↑)	0.84	(0.82–0.87)	16% (↓)
Radiotherapy	1.43	(1.27–1.58)	43% (↑)	1.19	(1.12–1.27)	19% (↑)	0.88	(0.83–0.92)	12% (↓)
Chemotherapy + Radiotherapy	0.82	(0.68–0.96)	18% (↓)	0.83	(0.74–0.93)	17% (↓)	0.84	(0.82–0.87)	16% (↓)
Surgery	0.82	(0.68–0.96)	18% (↓)	0.83	(0.77–0.89)	17% (↓)	0.99	(0.93–1.05)	1% (⥈)

↑: increase; ↓: decrease; ⥈: equal∗.

In the 31–60-day interval, Table 3 shows that ITARs were closer to unity for most modalities, indicating partial system adaptation. Radiotherapy continued to show elevated access (ITAR 1.19; 95% CI: 1.12–1.27), while chemotherapy slightly exceeded expectations (ITAR 1.04; 95% CI: 1.01–1.08). In contrast, surgery remained below expected levels (ITAR 0.83; 95% CI: 0.77–0.89), reflecting persistent constraints on surgical capacity. For treatments initiated after 60 days, ITARs generally approached expected levels, suggesting partial recovery. Nonetheless, chemotherapy (ITAR 0.84; 95% CI: 0.82–0.87) and surgery (ITAR 0.99; 95% CI: 0.93–1.05) continued to demonstrate residual underperformance, indicating that longer treatment delays remained unresolved for selected modalities.

## Discussion

This nationwide, population-based study provides the first comprehensive assessment of how the COVID-19 pandemic affected access to CRC treatment in Brazil, integrating spatial analysis with interrupted time-series modeling. The findings demonstrate substantial disruptions in timely treatment initiation, marked regional heterogeneity, and modality-specific responses that persisted beyond the acute phase of the pandemic. Together, these results highlight how pre-existing structural inequities in oncology care were amplified under conditions of systemic stress.

Marked geographic disparities in access to CRC treatment reflect long-standing inequalities in the territorial distribution of oncology services in Brazil. Health Regions in South and Southeast, particularly those with more developed urban infrastructure and specialized cancer centers, showed higher baseline access and greater resilience during the pandemic. These regions concentrate a larger share of Brazil's specialized cancer care facilities, benefit from better territorial organization of services.^4^^,^^26^ In contrast, North and Midwest experienced sharper declines, especially for high-complexity modalities such as surgery and chemotherapy. These disparities are influenced by vast geographic areas with low population density, logistical challenges in service distribution and a concentration of healthcare infrastructure in capitals, which disproportionately affect access for inland and remote populations.^27^^,^^28^

Although the formal adoption of Health Regions as a territorial intended to ensure equitable access within the SUS, the pandemic exposed persistent gaps in the operationalization of this model. The principle of territorialization, which is key to organizing regional care flows and reducing access barriers, was undermined by chronic underinvestment in regional capacity, workforce shortages, and limited redundancy in high-complexity services reduced the system's ability to absorb sudden shocks.^29^^,^^30^ Similar patterns have been observed in other large middle and high-income countries with universal health systems. In Canada and the UK, for example, studies have identified spatial inequalities in access to oncologic treatment, especially in remote or rural regions.^31^^,^^32^ Even in England, where the 62-day cancer treatment target policy has been in place, data suggest persistent regional variation in compliance, reflecting service delivery constraints and workforce shortages.^33^ However, Brazil's experience appears to have been exacerbated by weaker federal coordination and the absence of a nationally organized CRC screening program, limiting the system's capacity to buffer disruptions across the care continuum.^12^^,^^34^ In large and diverse health systems, effective territorial governance is critical to achieving equitable cancer care, particularly in times of systemic stress such as the COVID-19 public health emergency.

Differences between Brazil and other countries may also reflect the national pandemic response context. In India, chemotherapy for CRC decreased by 70% during the pandemic,^35^ while reductions of 17.1% and 11% were documented in China and the United Kingdom, respectively.^36^^,^^37^ Population-based analyses also confirmed this trend. In England, the volume of CRC surgeries has fallen by 31% relative to pre-pandemic expectations, whereas short-course radiotherapy procedures have increased by 41%, reflecting shifts in clinical prioritization.^38^ In this context, Brazil reported its first COVID-19 case in February 2020, with a rapid escalation of infections and deaths culminating in a peak in May of the same year. Notably, the absence of a coordinated federal strategy to manage the health crisis due to the discrediting of the pandemic through fake news and delays in the timely purchase of vaccines led to a fragmented response, with states and municipalities independently implementing containment measures, including lockdowns.^12^ National clinical bodies issued emergency guidance, such as the Brazilian Society of Coloproctology, which recommended in early 2020 that surgical interventions be limited to cases with curative intent, encouraging neoadjuvant approaches such as short-course radiotherapy instead.^8^ This shift in clinical decision-making may partly explain the observed increase in radiotherapy use during the pandemic period in Brazil.

The rise in radiotherapy use should therefore not be interpreted as improved access, but rather as an adaptive response to constrained surgical capacity. Radiotherapy is less dependent on inpatient beds and intensive care resources and can be delivered in shorter regimens, making it more feasible during periods of system stress.^39^^,^^40^ Conversely, surgical care is highly sensitive to operating room availability, anesthesia services, and postoperative intensive care, all of which were significantly reduced during COVID-19.^41^ Regional variability in surgical access further reflects differences in local hospital infrastructure and workforce availability, reinforcing the role of territorial capacity in shaping treatment pathways during crises.^42^

The COVID-19 pandemic disrupted CRC treatment protocols worldwide, prompting a shift from surgical interventions toward greater use of neoadjuvant therapies such as chemotherapy and radiotherapy. This trend is consistent with findings from recent international studies, including one that documented a widespread move toward non-surgical modalities during the pandemic period.^41^ In Brazil, national CRC treatment guidelines such as the Clinical Protocol and Therapeutic Guidelines for Oncology prioritize surgical resection, either as a primary intervention or following neoadjuvant chemotherapy.^43^ Similar treatment sequences are adopted in countries such as South Korea and Japan, where sharp reductions in surgical procedures for CRC were also observed during the pandemic.^44^^,^^45^ Together, these findings suggest that public health emergencies such as COVID-19 not only disrupt service capacity but also prompt strategic shifts in treatment sequencing, particularly in high-complexity modalities.

A significant decline in CRC screening and diagnostic testing were reported globally during the COVID-19 pandemic, contributing to delayed diagnoses and potential shifts in treatment patterns. An estimated 7.4 million fecal immunochemical tests (FIT) were not performed in 2020, potentially resulting in over 13,000 additional CRC cases projected through 2050.^46^ Overall, CRC screening rates decline by 44.3% across modalities,^47^ with more severe reductions reported in some countries such as the United States, where screening rates decreased by 79.3%, equating to 3.8 million missed tests in 2020 alone.^48^ In Brazil, the absence of a nationally implemented CRC screening program within the public healthcare system, coupled with fragmented, municipality-driven initiatives, has further limited access and hindered consistent data collection.^49^ These interruptions in screening and early detection are likely to have contributed to the patterns observed in our study, including the sharp decline in timely surgical interventions and the rise in treatment initiation beyond 60 days. As CRC diagnosis was delayed or deferred, treatment began at more advanced stages or after extended intervals, underscoring how upstream disruptions in screening cascade into significant downstream impacts on therapeutic access.

Our analysis has several important strengths. It leverages a nationwide, population-based dataset covering multiple years, allowing for robust temporal and spatial analyses of colorectal cancer treatment access across Brazil. The use of standardized indicators, interrupted time-series methods, and Health Region-level analyses enables the identification of regional disparities and modality-specific vulnerabilities during a major health system emergency. Nevertheless, some limitations should be considered. This study has limitations inherent to its ecological, population-based design, which precludes individual-level inference and direct extrapolation to clinical outcomes. To mitigate this, we used long time-series data, age-standardized rates, and complementary spatial and interrupted time-series analyses to strengthen population-level inference. Second, the reliance on administrative data from public oncology services within the Brazilian Unified Health System may underestimate national treatment patterns by excluding care delivered in the private sector; however, the public system provides the majority of colorectal cancer care in Brazil, supporting the public health relevance of these findings. Although data completeness improved after 2018 following changes in reporting protocols, diagnostic information prior to this period remains heterogeneous; this was addressed by emphasizing temporal changes rather than absolute levels. Data quality also varies across Health Regions, with more complete reporting in areas with established oncology centers, which was considered in the interpretation of spatial patterns. Finally, the absence of detailed sociodemographic and clinical variables, including race, ethnicity, and comorbidities, limited the assessment of differential impacts. Future studies integrating cancer registries, clinical datasets, and individual-level sociodemographic information are needed to better characterize inequities and pandemic-related disparities.

This study offers the first nationwide, population-based assessment of how the COVID-19 pandemic affected access to colorectal cancer treatment in Brazil across therapeutic modalities, treatment intervals, and health regions. From a public health perspective, these findings highlight structural weaknesses in the organization and regional distribution of oncology services within large, decentralized health system. The concentration of treatment disruptions in socioeconomically disadvantaged regions reinforces the need for territorial planning policies that prioritize equity, strengthen regional oncology care networks, and ensure surge capacity during health emergencies. Policy responses should focus on restoring and protecting surgical and systemic treatment capacity, improving coordination across levels of care, and integrating contingency planning into national cancer control strategies. In addition, the differential patterns observed across treatment modalities emphasize the importance of modality-specific monitoring and adaptive service delivery models. Sustained investment in population-based surveillance systems is essential to track recovery trajectories, identify persistent bottlenecks, and support data-driven decision-making. Strengthening early detection pathways and safeguarding timely treatment access must remain central priorities to prevent future crises from exacerbating existing disparities in colorectal cancer care.

## Contributors

Conceptualization: LRSM, JSP, ADS. Data curation: LRSM, JSP, ADS. Formal analysis: LRSM, JSP. Investigation: LRSM, JSP. Methodology: LRSM, JSP, ADS. Project administration: LRSM, JSP, ADS. Resources: LRSM, JSP, ADS, CAL. Supervision: ADS. Visualization: LRSM, JSP, ADS, CAL. Writing—original draft: LRSM, JSP. Writing—review & editing: ADS, CAL, CJNR, CDFS, MSM.

LRSM, JSP, MMS, and ALS verified the underlying data used in the analyses. LRSM, JSP and ALS had full access to the raw data and took responsibility for the integrity of the data and the accuracy of the analyses. Final responsibility for the decision to submit for publication: all authors.

## Data sharing statement

All data used in this study are publicly available and can be accessed through national open-access databases maintained by the Brazilian Ministry of Health and the Brazilian Institute of Geography and Statistics (IBGE). Specifically, health-related data were obtained from the Information Technology Department of the Unified Health System (DATASUS), available at: http://www.datasus.gov.br. Population estimates and geographical data (including shapefiles of health regions) were retrieved from the IBGE platform, accessible at: https://www.ibge.gov.br. Data collection, maintenance, and quality assurance are the responsibility of these official governmental agencies. The authors did not have any privileged access to these databases, and all information used in the analysis is openly available to the public and the scientific community.

## Editor note

The Lancet Group takes a neutral position with respect to territorial claims in published maps and institutional affiliations.

## Generative AI statement

During the preparation of this work, the authors used ChatGPT (GPT-4, OpenAI, accessed via ChatGPT Plus, May 2025) to improve the clarity, grammar, and fluency of the English language. Specifically, the tool was employed to rephrase complex sentences, enhance linguistic style, and improve coherence. No large language model was used to generate original scientific insights, analyze or interpret data, or draw conclusions. The final manuscript was carefully reviewed by all authors to ensure that the meaning, scientific accuracy, and conclusions remained unchanged. After using this tool, the authors reviewed and edited the content as needed and took full responsibility for the content of the publication.

## Declaration of interests

We declare no competing interests.

## References

[1] Bray F., Laversanne M., Sung H. (2024). Global cancer statistics 2022: GLOBOCAN estimates of incidence and mortality worldwide for 36 cancers in 185 countries. CA Cancer J Clin.

[2] Khan S.Z., Lengyel C.G. (2023). Challenges in the management of colorectal cancer in low- and middle-income countries. Cancer Treat Res Commun.

[3] (2022). Instituto Nacional do Câncer José Alencar Gomes da Silva (INCA), Estimativas 2023: Incidência de Câncer no Brasil, Rio de Janeiro.

[4] Fonseca B.P., Albuquerque P.C., Saldanha R.F., Zicker F. (2022). Geographic accessibility to cancer treatment in Brazil: a network analysis. Lancet Reg Health Am.

[5] Oliveira N.P., Mendes T.M.C., Vasconcelos H.S., Castro J.L., Souza D. (2025). Oncological care network: spatial distribution of resources and health workforce in Brazil. Rev Bras Cancerol.

[6] Villarreal-Garza C., Aranda-Gutierrez A., Gonzalez-Sanchez D. (2025). National cancer control plans in Latin America and the Caribbean: challenges and future directions. Lancet Oncol.

[7] Mazidimoradi A., Hadavandsiri F., Momenimovahed Z., Salehiniya H. (2023). Impact of the COVID-19 pandemic on colorectal cancer diagnosis and treatment: a systematic review. J Gastrointest Cancer.

[8] Campos F.G., Sarubbi Fillmann H. (2020). General recommendations to the colorectal surgeon during the COVID-19 pandemic. J Coloproctol.

[9] Freund M.R., Wexner S.D. (2022). Trends in colorectal surgery during the COVID-19 pandemic. JAMA Netw Open.

[10] Durán D., Anyosa R.C., Nicolau B., Kaufman J.S. (2023). Uncovering the impact of COVID-19 on the place of death of cancer patients in South America. Cad Saude Publica.

[11] Fonseca G.A., Normando P.G., Loureiro L.V.M. (2021). Reduction in the number of procedures and hospitalizations and increase in cancer mortality during the COVID-19 pandemic in Brazil. JCO Glob Oncol.

[12] Ribeiro C.M., Atty A.T.M. (2025). The impact of the COVID-19 pandemic on cancer care in Brazil: from screening to treatment. Rev Bras Cancerol.

[13] Oliveira H.F., Yoshinari G.H., Veras I.M. (2022). Impact of the COVID-19 pandemic on radiation oncology departments in Brazil. Adv Radiat Oncol.

[14] Hartman H.E., Sun Y., Devasia T.P. (2020). Integrated survival estimates for cancer treatment delay among adults with cancer during the COVID-19 pandemic. JAMA Oncol.

[15] Brasil (2012). https://www.planalto.gov.br/ccivil_03/_ato2011-2014/2012/lei/l12732.htm.

[16] Elamin D., Ozgur I., Steele S.R., Khorana A.A., Jia X., Gorgun E. (2023). Impact of COVID-19 pandemic on treatment of colorectal cancer patients. Am J Surg.

[17] Brasil. Ministério da Saúde. Secretaria de Atenção Especializada à Saúde (2024). Relatório preliminar do Protocolo Clínico e Diretrizes Terapêuticas (PCDT) do adenocarcinoma de cólon e reto.

[18] (2025). Instituto Brasileiro de Geografia e Estatística (IBGE), Portal Cidades.

[19] Brasil (2011). http://www.planalto.gov.br/ccivil_03/_ato2011-2014/2011/decreto/D7508.htm.

[20] (2024). Departamento de Informação e Informática do Sistema Único de Saúde (DATASUS) do Ministério da Saúde, Informações de Saúde, Distrito Federal.

[21] Segi M. (1960).

[22] Doll R., Payne P., Waterhouse J. (1966).

[23] Microsoft Corporation (2025).

[24] QGIS (2021). QGIS geographic information system. https://qgis.org/.

[25] (2025). RStudio Team, RStudio: Integrated Development for R. https://www.rstudio.com.

[26] Saldanha R.F., Xavier D.R., Carnavalli K.M., Lerner K., Barcellos C. (2019). Analytical study of the breast cancer patient flow network in Brazil from 2014 to 2016. Cad Saude Publica.

[27] da Silva M.J.S., O'Dwyer G., Osorio-de-Castro C.G.S. (2019). Cancer care in Brazil: structure and geographical distribution. BMC Cancer.

[28] Strasser-Weippl K., Chavarri-Guerra Y., Villarreal-Garza C. (2015). Progress and remaining challenges for cancer control in Latin America and the Caribbean. Lancet Oncol.

[29] Iosti P. (2020). Territorialization of care and proximities in a community-based primary care system: what are the results on access to care and resident satisfaction? A case study from São Paulo. Health Place.

[30] Botega L.A., Andrade M.V., Guedes G.R., Nogueira D. (2022). Spatial reorganization of the Brazilian Unified National Health System's inpatient care supply. Cad Saude Publica.

[31] Chan J., Polo A., Abdel-Wahab M., Hirata D., Bourque J.M., Zubizarreta E. (2018). Disparities in accessibility to radiation therapy in a high-income country: the case of Canada. Int J Radiat Oncol Biol Phys.

[32] Møller H., Coupland V.H., Tataru D. (2018). Geographical variations in the use of cancer treatments are associated with survival of lung cancer patients. Thorax.

[33] Cancer Research UK (2025). https://news.cancerresearchuk.org/2025/04/10/cancer-waiting-times-latest-updates-and-analysis/.

[34] Boschiero M.N., Palamim C.V.C., Ortega M.M., Mauch R.M., Marson F.A.L. (2021). One year of coronavirus disease 2019 (COVID-19) in Brazil: a political and social overview. Ann Glob Health.

[35] Raj Kumar B., Pandey D., Rohila J., deSouza A., Saklani A. (2020). An observational study of the demographic and treatment changes in a tertiary colorectal cancer center during the COVID-19 pandemic. J Surg Oncol.

[36] Xu Y., Huang Z.H., Zheng C.Z. (2021). The impact of COVID-19 pandemic on colorectal cancer patients: a single-center retrospective study. BMC Gastroenterol.

[37] Merchant J., Lindsey I., James D. (2021). Maintaining standards in colorectal cancer surgery during the global pandemic: a cohort study. World J Surg.

[38] Morris E.J.A., Goldacre R., Spata E. (2021). Impact of the COVID-19 pandemic on the detection and management of colorectal cancer in England: a population-based study. Lancet Gastroenterol Hepatol.

[39] Vecchione L., Stintzing S., Pentheroudakis G., Douillard J.Y., Lordick F., ESMO Guidelines Committee (2020). ESMO management and treatment adapted recommendations in the COVID-19 era: colorectal cancer. ESMO Open.

[40] Mukherji R., Marshall J.L. (2021). Lessons learned in managing patients with colorectal cancer during the COVID-19 pandemic. Curr Treat Options Oncol.

[41] Pararas N., Pikouli A., Papaconstantinou D. (2022). Colorectal surgery in the COVID-19 era: a systematic review and meta-analysis. Cancers (Basel).

[42] Moszkowicz L.B.D.M., Gonçales T.A., Debastiani M.S. (2025). Impact of the COVID-19 pandemic on the outcomes of colorectal cancer surgical patients treated at a public hospital in Southern Brazil. Front Surg.

[43] Brasil. Ministério da Saúde. Secretaria de Atenção à Saúde (2014). https://pesquisa.bvsalud.org/portal/resource/pt/sms-9812.

[44] Fujita M., Yamaguchi K., Nagashima K. (2023). Changes in colorectal cancer treatment during the COVID-19 pandemic in Japan: interrupted time-series analysis using the National Database of Japan. Cancer Epidemiol.

[45] Choi J., Park I., Lee H. (2021). Impact of the COVID-19 pandemic on surgical treatment patterns for colorectal cancer in a tertiary medical facility in Korea. Cancers.

[46] Nodora J., Gupta S., Howard N. (2021). The COVID-19 pandemic: identifying adaptive solutions for colorectal cancer screening in underserved communities. J Natl Cancer Inst.

[47] Teglia F., Angelini M., Astolfi L., Casolari G., Boffetta P. (2022). Global association of COVID-19 pandemic measures with cancer screening: a systematic review and meta-analysis. JAMA Oncol.

[48] Chen R.C., Haynes K., Du S., Barron J., Katz A.J. (2021). Association of cancer screening deficit in the United States with the COVID-19 pandemic. JAMA Oncol.

[49] Toledo C., Almeida L.M.P.R., Averbach M., Barbosa J. (2023). Analysis of the tracking initiatives of colorectal cancer in Brazil. Arq Gastroenterol.

